# Prevalence of Smartphone Addiction and Its Associated Factors Among College Students Aged 18-23 Years in Kerala, India: A Cross-Sectional Study

**DOI:** 10.7759/cureus.97301

**Published:** 2025-11-20

**Authors:** Manuja Devi, Chitra Tomy, Jeby Jose Olickal, Brilly Michael Rose, Kavumpurathu Raman Thankappan

**Affiliations:** 1 Department of Public Health, Amrita Institute of Medical Sciences, Amrita Vishwa Vidyapeetham, Kochi, IND; 2 Department of Community Medicine, Amrita Institute of Medical Sciences, Amrita Vishwa Vidyapeetham, Kochi, IND

**Keywords:** cross-sectional study, mental health, problematic smartphone use, smartphone addiction, university students

## Abstract

Background

Smartphone addiction is a growing public health concern, especially among young adults. Literature regarding the prevalence and associated factors of smartphone addiction among young adults in Kerala remains limited and inconsistent. Therefore, this study aimed to estimate the prevalence of smartphone addiction and to identify its associated factors among college students in Kerala.

Methods

We conducted a cross-sectional study among 1,067 college students aged 18-23 years (53% females) from Thiruvananthapuram and Thrissur districts of Kerala. Students from government, aided, and self-financing colleges were selected through a multistage cluster sampling method, where colleges and classes were randomly chosen. This approach helped ensure that the sample was representative and minimized selection bias, giving all eligible students in the selected clusters an equal chance of participation. The study was conducted over a period of nine months, from August 2024 to May 2025, with data collected between December 20, 2024, and February 20, 2025, using the Mobile Phone Use Screening Test (MUST) questionnaire. Data were analyzed using jamovi (Version 2.3) ([Computer Software]. Retrieved from https://www.jamovi.org)) for descriptive statistics, and multivariable logistic regression was performed using IBM SPSS Statistics for Windows, V. 20.0 (IBM Corp., Armonk, NY, USA).

Results

The prevalence of smartphone addiction was 39.9% (95% CI: 37-42.9). Factors significantly associated with smartphone addiction included a delay of over 30 minutes between phone use and sleep (aOR = 3.38; 95% CI: 1.84-6.19), smartphone use before sleep (aOR = 2.96; 95% CI: 1.80-4.86), use after midnight (aOR = 1.93; 95% CI: 1.44-2.57), being from a nuclear family (aOR = 1.55; 95% CI: 1.11-2.17), and using the phone for ≥30 minutes after lights off without a blue light filter (aOR = 1.48; 95% CI: 1.14-1.93).

Conclusion

The study reveals a high prevalence of smartphone addiction among college students in Kerala. Targeted interventions addressing smartphone addiction among college students in Kerala are critical, especially for those from nuclear families and those who use their devices late at night. These at-risk groups include students who use smartphones after midnight, use them for 30 minutes or more after turning off the lights, or have a delay between smartphone use and falling asleep.

## Introduction

Smartphone addiction is conceptualized as a behavioral addiction characterized by features such as mood tolerance, salience, withdrawal, modification, conflict, and relapse. The development of multifunctional smartphones and their widespread global popularity have significantly transformed the communication and information landscape worldwide. While these devices reshaped users' interests, values, and desires, they have also raised significant global concerns about overuse and addiction [1]. A meta-analysis estimated the global pooled prevalence of smartphone addiction to be 26.99% [2]. Over the past decade, smartphone ownership has increased dramatically, with more than half of the global population owning the device. In high-income countries such as North America and Europe, more than 80% of the population owns a smartphone, while ownership rates in low- and middle-income countries continue to increase [3]. The number of smartphone users worldwide is estimated to be 5.61 billion, accounting for 69.4% of the global population, and is expected to increase further in the coming years [4].

Recent international studies have identified a range of psychological and social factors that drive excessive smartphone use. A Lebanese study found that "boredom, loneliness, anxiety, stress, and depression increase the propensity to develop smartphone addiction" [5]. Similarly, in China, college students with greater anxiety (and poor sleep quality) were far more likely to exhibit problematic phone use [6]. Further, socioeconomic and lifestyle factors play a role: a study in Tibet revealed that higher income, higher education level, and younger age were associated with excessive use, while regular physical exercise had a protective effect. Importantly, factors beyond the individual also play a role [7]. In the context of low‑ and middle‑income countries, bedtime procrastination mediated the pathway from smartphone overuse to negative health outcomes, emphasizing how broken sleep patterns may both drive and result from excessive usage [8]. Together, these findings suggest that both personal emotional distress and external social pressures contribute to excessive smartphone use worldwide.

Young people are the most vulnerable group to develop problematic smartphone use. A survey by the Mobile Ecosystem Forum reported the highest smartphone penetration among individuals aged 16-24 years, with a prevalence of 37% [9]. Within this younger population, 22% reported checking their smartphones every few minutes, and nearly half of parents (47%) believed their children were addicted to their devices [10]. These patterns highlight the growing risk of addiction among young adults. In India, regional studies reported high prevalence rates of smartphone addiction among students, with 46.15% in Maharashtra and 35.25% in Kerala [11,12]. Such findings emphasize the considerable burden of smartphone addiction among young adults in different parts of the country, particularly in Kerala. In addition, socio-demographic and usage-related variables such as male gender, higher family income, lack of parental supervision, excessive social media use, entertainment and gaming activities, and underlying psychological distress were associated with heightened risk [13]. The risky use of smartphones negatively affects daily activities. Education and counselling programs were suggested in the literature to prevent smartphone addiction among students [14].

Although smartphone addiction has been recognized as a major global concern, research focusing on its prevalence and associated factors among young adults aged 18-23 years remains limited in India. Inconsistencies in measurement tools and definitions further hinder accurate assessment and cross-study comparisons, highlighting the need for standardized approaches. Kerala, with its high literacy rate and extensive smartphone penetration, provides a relevant setting for studying this issue. Therefore, this study aims to determine the prevalence of smartphone addiction among college students in Kerala using the standardized Mobile Phone Use Screening Test (MUST) questionnaire and to identify the factors associated with smartphone addiction, including smartphone usage habits such as duration, frequency, and timing, particularly before sleep.

## Materials and methods

Study design and setting

An institution-based cross-sectional study was conducted in Kerala, India, over a nine-month period from August 2024 to May 2025, with data collection carried out between December 20, 2024, and February 20, 2025. Two districts, Thiruvananthapuram and Thrissur, were randomly selected using the lottery method: one from the northern seven districts and another from the southern seven districts. Colleges were identified among programs affiliated with the University of Kerala, the University of Calicut, and the All India Council for Technical Education. The selected institutions included government, aided (private colleges with government support), and self-financing colleges from the Arts and Science stream, as well as government and self-financing colleges from the Engineering sector.

Study population

This study was conducted among young adults aged 18-23 years enrolled in selected colleges in Kerala, India.

Inclusion and exclusion criteria

Inclusion Criteria

College students aged 18-23 years who were residents of Kerala were included in the study.

Exclusion Criteria

Students with sleep disorders unrelated to smartphone use were excluded. Before distributing the questionnaire, the respective class teachers were consulted to identify if any students in their class had such conditions.

Sample size

Based on the cross-sectional study by Dharmadhikari et al. [11], which reported a smartphone addiction prevalence of 46.15%, the required sample size was calculated using the formula $n = \frac{z^2_{\frac{\alpha}{2}} p(1-p)}{d^2}$, where Z(1−𝛼/2) = 1.96 for a 95% confidence level, p = 0.46, and d = 0.05 (absolute error); the calculated sample size was 382. With a design effect of 2 to account for the cluster sampling design and a 20% non-response rate, the final sample size of the study was 955, which was rounded off to 1000.

Sampling technique

A multistage cluster sampling technique was used for this study. The sample was allocated to achieve a fixed total of 1,000 students, with 600 from Arts and Science colleges and 400 from Engineering colleges. In the first stage, colleges were stratified by type (government, aided, and self-financing for Arts and Science; government and self-financing for Engineering) within each district. Using computer-generated random numbers, three Arts and Science colleges (one government, one aided, and one self-financing) and two Engineering colleges (one government and one self-financing) were selected from each district.

In the second stage, classrooms within the selected colleges were considered as the primary sampling units (clusters). From each Engineering college, two classes (anticipated size 45-55 students per class) were randomly chosen, while from each Arts and Science college, four classes (anticipated size 20-30 students per class) were selected using simple random sampling. All eligible students in the chosen classes were invited to participate.

**Figure 1:**
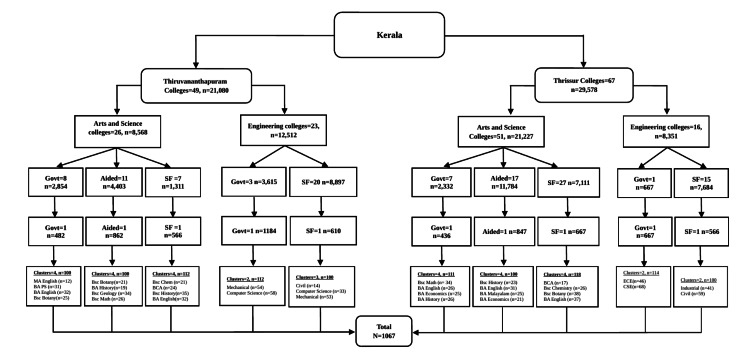
Flowchart showing the selection of college students Govt: government; SF: self-financing college; MA: Master of Arts; BA: Bachelor of Arts; BSc: Bachelor of Science; BCA: Bachelor of Computer Applications; CSE: Computer Science and Engineering; ECE: Electronics and Communication Engineering

In total, 33 classroom clusters were included in the study: 24 from Arts and Science colleges and nine from Engineering colleges. In one self-financing Engineering college in Thiruvananthapuram, an additional class was sampled because the required number of participants could not be obtained from the initially selected clusters. The final sample comprised 1,067 students from 10 colleges affiliated to the University of Kerala and the University of Calicut (Figure 1).

Study tools and procedure

Data were collected using a semi-structured, validated questionnaire comprising two sections (see Appendices). The first section, which consisted of socio-demographic variables and smartphone usage patterns, was developed by the authors for the specific context of this study. The second section included the MUST, a validated tool developed by Sharma et al. [15] at the Service for Healthy Use of Technology (SHUT) Clinic, National Institute of Mental Health and Neurosciences, Bengaluru, which evaluates excessive or addictive smartphone use. MUST consists of 18 items rated on a 5-point Likert scale ranging from "Never" to "Always". The scale has four components: craving, loss of control, coping, and consequences. Smartphone use was classified as mild use (≤30), moderate use (31-49), and excessive/addictive use (≥50). The tool has demonstrated good reliability, with a Cronbach's alpha of 0.93 and split-half reliability of 0.86 [15]. Permission to use MUST was obtained from the respective developers. Data collection was carried out by the lead author (MD) from December 2024 to February 2025. Data were collected using Epicollect, a secure mobile-based platform, and subsequently exported to Microsoft Excel (Microsoft Corp., Redmond, WA, USA) for cleaning and verification. The data cleaning process involved checking for missing values, inconsistencies, and duplicates to ensure accuracy and completeness prior to analysis.

Permission from the principals of each college was obtained before data collection. Participation was voluntary after obtaining written informed consent. Questionnaires were distributed in classrooms, and a brief explanation of the study's general purpose was provided to students who consented to participate. All the students present in the class during the time of data collection consented to participate in the study.

Statistical analysis

The data collected were entered into Microsoft Excel and analyzed using jamovi (Version 2.3) ([Computer Software]. Retrieved from https://www.jamovi.org)). Quantitative variables were summarized as means and standard deviations, while qualitative variables were presented as frequencies and percentages. The prevalence of smartphone addiction was reported as frequency and percentage with a 95% confidence interval. The chi-squared test or Fisher's exact test was used to identify significant associations between smartphone addiction and independent variables. Multivariable logistic regression analysis was performed to identify factors associated with smartphone addiction, and collinearity among predictors was assessed using the variance inflation factor (VIF) values. A p-value of <0.05 was considered statistically significant.

Ethical considerations

Ethical approval for this study was obtained from the Ethics Committee of Amrita School of Medicine (approval number: ECASM-AIMS-2024-597). Permission to conduct the study was also obtained from the principals of all participating colleges. Participation was voluntary after obtaining written informed consent from all students. Questionnaires were distributed in classrooms, and a brief explanation of the study's general purpose was provided to those who consented to participate. Confidentiality was maintained throughout the study.

## Results

Table 1 presents the demographic and smartphone usage characteristics of the 1,067 college students surveyed. The median age of participants was 20 years (interquartile range (IQR): 19-21), with 712 (66.7%) aged ≥20 years and 569 (53.3%) females. Most participants were Hindu (610, 57.2%) and above the poverty line (740, 69.4%). The majority were single (1,058, 99.2%), living with their family (781, 73.2%), and from nuclear families (880, 82.5%). The median age at which participants began using smartphones was 15 years (IQR: 9-21). Daily smartphone use of 5-6 hours was reported by 474 (44.4%) students, with social media being the primary purpose for 541 (50.7%) participants. Notably, 934 (87.5%) participants used smartphones before going to sleep, 802 (75.2%) used them within 30 minutes of bedtime, and 565 (53%) used them after lights out without blue light filters. 

**Table 1:** Distribution of socio-demographic characteristics, smartphone usage patterns, perceived addiction, and sleep habits among the study participants (n = 1,067) APL: above poverty line; BPL: below poverty line

Variable	Category	Frequency (n)	Percentage (%)
Course	Arts and Science	642	60.2
	Engineering	425	39.8
Age (in years)	<20	355	33.3
	≥20	712	66.7
Gender	Female	569	53.3
	Male	490	45.9
	Others	8	0.8
Religion	Hindu	610	57.2
	Christian	279	26.1
	Muslim	178	16.7
Marital status	Single	1,058	99.2
	Married	9	0.8
Way of living	Hosteler	286	26.8
	With family	781	73.2
Socioeconomic status	APL	740	69.4
	BPL	327	30.6
Type of family	Nuclear	880	82.5
	Joint	153	14.3
	Three-generational family	34	3.2
Age of initiation of smartphone use	<15 years	561	52.6
	≥15 years	506	47.4
Daily duration of smartphone use (in hours)	Less than 1 hour	36	3.4
	2 hours	97	9.1
	3 hours	249	23.3
	5-6 hours	474	44.4
	7-8 hours	131	12.3
	More than 8 hours	80	7.5
The primary purpose of smartphone use (multiple options)	Reading	22	2.1
	Songs	89	8.3
	Messaging	69	6.5
	Internet/browsing	116	10.8
	Phone calls	34	3.2
	Social media	541	50.7
	Watching videos	196	18.4
Smartphone use right before sleeping	Yes	934	87.5
	No	133	12.5
Latest time of smartphone use before sleep	Before 11:00 pm	308	28.9
	11:00 pm	126	11.8
	11:30 pm	144	13.5
	12:00 am	192	18
	12:30 am	154	14.4
	1:00 am or later	143	13.4
Time spent on smartphone before sleep (minutes/hours)	<30 minutes	802	75.2
	30 minutes to 1 hour	206	19.3
	>1 hour	59	5.5
Smartphone use for at least 30 minutes after lights off without a blue light filter	Yes	565	53
	No	502	47

Table 2 shows the distribution of responses to the MUST questionnaire among participants. The responses are scored from 1 (never) to 5 (always), reflecting the frequency of smartphone use, dependency, and its effects on daily activities, relationships, and sleep. The most common behaviors were starting the day with smartphone use (301, 28.2%), feeling uncomfortable without a smartphone (250, 23.4%), experiencing problems in college/work due to smartphone use (143, 13.4%), difficult to stop using the smartphone (336, 31.5%), and using a smartphone to feel good (254, 23.8%).

**Table 2:** Distribution of responses to the MUST questionnaire among the participants (n = 1,067) MUST: Mobile Phone Use Screening Test

Sl. no.	Question	Never, n (%)	Rarely, n (%)	Occasionally, n (%)	Frequently, n (%)	Always, n (%)
1	I start my day with the use of a smartphone	146 (13.7%)	235 (22%)	301 (28.2%)	143 (13.4%)	242 (22.7%)
2	I wish I had my smartphone in my hand round the clock	340 (31.9%)	228 (21.4%)	263 (24.6%)	132 (12.4%)	104 (9.7%)
3	I am uncomfortable without my smartphone	274 (25.7%)	310 (29.1%)	250 (23.4%)	121 (11.3%)	112 (10.5%)
4	I can manage the day without my smartphone	223 (20.8%)	215 (20.1%)	242 (22.7%)	191 (17.8%)	196 (18.6%)
5	I experience problems in school, college, or work due to excessive use of smartphones	521 (48.8%)	231 (21.6%)	173 (16.2%)	81 (7.6%)	61 (5.7%)
6	I experience problems in college/workplace due to the usage of smartphone	604 (56.7%)	221 (20.6%)	143 (13.4%)	66 (6.2%)	33 (3.1%)
7	I experience problems in relationships due to my preoccupation with smartphone	504 (47.2%)	266 (24.9%)	168 (15.7%)	80 (7.5%)	49 (4.6%)
8	I can reduce the usage of my smartphone	93 (8.7%)	181 (17%)	245 (23%)	278 (26%)	270 (25.3%)
9	I experience sleep disturbance due to late-night usage of smartphone	294 (27.6%)	300 (28%)	261 (24.5%)	131 (12.3%)	81 (7.6%)
10	Reduction of battery power on my smartphone disturbs me	240 (22.5%)	255 (23.9%)	289 (27.1%)	190 (17.8%)	93 (8.7%)
11	Whenever I have a smartphone with me, it is difficult to stop using it	170 (15.9%)	284 (26.6%)	336 (31.5%)	180 (16.9%)	97 (9.1%)
12	I indulge in smartphone usage for longer than earlier	116 (10.9%)	231 (21.6%)	317 (29.7%)	239 (22.4%)	164 (15.4%)
13	I use a smartphone to feel good	104 (9.7%)	207 (19.4%)	320 (30%)	254 (23.8%)	182 (17.1%)
14	Smartphone use helps me to overcome my stress	118 (11.1%)	237 (22.2%)	302 (28.3%)	241 (22.6%)	169 (15.8%)
15	I feel more confident while using my smartphone	194 (18.2%)	239 (22.4%)	331 (31%)	173 (16.2%)	130 (12.2%)
16	I use my smartphone as the only enjoyable activity of the day	240 (22.5%)	244 (22.9%)	316 (29.6%)	155 (14.5%)	112 (10.5%)
17	When I can't use my smartphone, I feel like I have lost my connection with others	333 (31.2%)	286 (26.8%)	246 (23%)	132 (12.4%)	70 (6.6%)
18	I use smartphones to escape or avoid day-to-day problems	302 (28.3%)	264 (24.7%)	281 (26.4%)	125 (11.7%)	95 (8.9%)

The median MUST score was 47 (IQR: 40-55). More than half of the students (606, 56.8%; 95% CI: 53.7-59.8) were moderate smartphone users, 426 (39.9%, 95% CI: 36.9-42.9) were excessive/addictive users, and 35 (3.3%) were mild users. Table 3 presents the factors associated with smartphones by bivariate analysis. Excessive use was more common among Engineering students (44.2%) than among Arts and Science students (37.1%), hostel residents (46.9%) than those living with family (7.4%), students belonging to above poverty line (APL) (41.9%) than below poverty line (BPL) (35.5%), and students from nuclear families (41.7%) than those from non-nuclear families (31.6%). Using a smartphone for ≥3 hours daily was strongly associated with smartphone addiction (42.1% vs. 24.8%; p < 0.001). Specific usage behaviors like using the phone before sleep (43.3% vs. 16.5%), after midnight (55.9% vs. 33.8%), within 30 minutes before sleep (67.8% vs. 38.3%), and after lights off without a blue light filter (46.2% vs. 32.9%) were also significant predictors of smartphone addiction. There was no collinearity between the variables used to assess smartphone use at bedtime. 

**Table 3:** Factors associated with smartphone addiction: results of bivariate analysis (n = 1067) P-values were calculated using the chi-squared test or Fisher's exact test. APL: above poverty line; BPL: below poverty line

Variable	Category	Excessive/addictive use	Mild/moderate use	Chi-square	P-value
Course	Arts and Science	238 (37.1%)	404 (62.9%)	5.47	0.01
	Engineering	188 (44.2%)	237 (55.8%)		
Age (in years)	<20 years	154 (43.4%)	201 (56.6%)	2.65	0.104
	≥20 years	272 (38.2%)	440 (61.8%)		
Gender	Female	210 (36.9%)	359 (63.1%)	5.87	0.053
	Male	211 (43.1%)	279 (56.9%)		
	Others	5 (62.5%)	3 (37.5%)		
Religion	Hindu	232 (38%)	378 (62%)	2.13	0.334
	Christian	118 (42.3%)	161 (57.7%)		
	Muslim	76 (42.7%)	102 (57.3%)		
Marital status	Single	425 (40.2%)	633 (59.8%)	3.14	0.07
	Married	1 (11.1%)	8 (88.9%)		
Way of living	Hosteler	134 (46.9%)	152 (53.1%)	7.82	0.005
	With family	292 (37.4%)	489 (62.6%)		
Socioeconomic status	APL	310 (41.9%)	430 (58.1%)	3.89	0.048
	BPL	116 (35.5%)	211 (64.5%)		
Type of family	Nuclear family	367 (41.7%)	513 (58.3%)	6.63	0.010
	Non-nuclear family	59 (31.6%)	128 (68.4%)		
Age of initiation of smartphone use	≤15 years	224 (39.7%)	337 (60.3%)	0.275	0.988
	>15 years	202 (39.9%)	304 (60.1%)		
Daily duration of smartphone use (hours)	≤2 hours	33 (24.8%)	100 (75.2%)	14.5	<0.001
	≥3 hours	393 (42.1%)	541 (57.9%)		
Smartphone use right before sleeping	Yes	404 (43.3%)	530 (56.7%)	34.6	<0.001
	No	22 (16.5%)	111 (83.5%)		
The latest time of smartphone use	On or before 12:00 am	260 (33.8%)	510 (66.2%)	43.7	<0.001
	After 12:00 am	166 (55.9%)	131 (44.1%)		
Time spent on smartphone before sleep (in minutes/hours)	≤30 minutes	386 (38.3%)	622 (61.7%)	20.2	<0.001
	>30 minutes	40 (67.8%)	19 (32.2%)		
Smartphone use for at least 30 minutes after lights off without a blue light filter	Yes	261 (46.2%)	304 (53.8%)	19.7	<0.001​​​​​​​
	No	165 (32.9%)	337 (67.1%)		

Table 4 represents the multivariable logistic regression analysis of factors associated with smartphone addiction. Participants who spent more than 30 minutes up to one hour on their smartphone before sleep had significantly higher odds of smartphone addiction (aOR = 3.38; 95% CI: 1.84-6.19; p < 0.001), indicating that prolonged pre-sleep smartphone use increases the risk of addictive behaviors. Students who used smartphones immediately before sleep also had higher odds of addiction (aOR = 2.96; 95% CI: 1.80-4.86; p < 0.001). Similarly, smartphone use after midnight was associated with greater odds (aOR = 1.93; 95% CI: 1.44-2.57; p < 0.001). Students from nuclear families were more likely to be addicted compared to those from non-nuclear families (aOR = 1.55; 95% CI: 1.11-2.17; p = 0.021). In addition, using the phone for ≥30 minutes after lights out without a blue light filter was significantly associated with smartphone addiction (aOR = 1.48; 95% CI: 1.14-1.93; p = 0.004). 

**Table 4:** Factors associated with excessive smartphone addiction: results of multivariable analysis (n = 1,067) Multivariable logistic regression was used to calculate the p-value. Collinearity diagnostics indicated no multicollinearity among the predictors. The highest VIF was observed for the latest time of smartphone use (VIF = 1.055), followed by the use of a smartphone for ≥30 minutes after lights off without a blue light filter (VIF = 1.046), smartphone use before sleep (VIF = 1.043), and use after midnight (VIF = 1.016). The highest condition index was 17.411, with no variance proportions ≥0.5 for multiple variables in the same dimension. AOR: adjusted odds ratio; CI: confidence interval; VIF: variance inflation factor

Variables	Category	AOR	95% CI for AOR		P-value
			Lower	Upper	
Type of family	Non-nuclear family	1	-	-	-
	Nuclear family	1.552	1.109	2.172	0.010
Smartphone use right before sleeping	No	1	-	-	-
	Yes	2.955	1.798	4.856	<0.001
Way of living	With family	1	-	-	-
	Hosteler	1.307	0.977	1.746	0.071
The latest time of smartphone use before sleep	On or before 12:00 am	1	-	-	-
	After 12:00 am	1.925	1.443	2.568	<0.001
Daily duration of smartphone use in hours	≤2 hours	1	-	-	-
	≥3 hours	1.536	0.986	2.392	0.058
Time spent on smartphone before sleep (in minutes/hour)	≤30 minutes	1	-	-	-
	>30 minutes	3.377	1.844	6.186	<0.001
Smartphone use for at least 30 minutes after lights off without a blue light filter	No	1	-	-	-
	Yes	1.481	1.136	1.931	0.004

## Discussion

The prevalence of smartphone addiction in this study was 39.9%, which is higher than the global pooled prevalence of 26.99% [2]. However, prevalence is lower than the national estimate of 46.15% [11] but similar to the previously reported prevalence of 35.25% from Kerala [12]. Globally, prevalence estimates vary widely, ranging from 2.4% to 60.3% [16,17]. Studies have reported varying prevalence rates of smartphone addiction, with 49.5% among medical students in China, 61.4% among young adults in Bangladesh, and 37.9% among medical students in Palestine [18-20]. This difference can be understood in the context of several contributing factors, like the population studied and the assessment tools used. Kerala has one of the highest literacy rates and internet penetration levels in India, which makes smartphones more accessible and widely used among young adults [21]. In addition, smartphone applications are intentionally designed to be engaging, with features such as instant notifications, social feedback, and infinite scrolling, which can lead to addictive behaviors [22]. A meta-analysis covering 19 studies conducted between 2014 and 2019 among medical students across seven Asian countries reported a pooled smartphone addiction rate of 41.93%, with factors including gender, daily usage duration, smartphone functions, and marital status influencing addiction levels [18].

In the present study, the strongest predictor of smartphone addiction was a delay of more than 30 minutes between smartphone use and sleep. Smartphone usage patterns like smartphone use before sleep, use after midnight, and using the phone for ≥30 minutes after lights off without a blue light filter were significant predictors of smartphone addiction. A study conducted among medical students reported that 94.3% of addicted users were using smartphones before sleep [11].

In our study, the odds of excessive smartphone addiction were higher among students from nuclear families than those from non-nuclear families. Students living in extended, three-generation, or joint families may experience more structured routines, which could reduce the risk of excessive smartphone use. The type of family is also important in our context since most students were living with their families. The relationship between family structure and smartphone addiction among young adults has been explored in several studies. A study from Bangladesh reported that 61.4% of young adults exhibited smartphone addiction, with individuals from larger families (≥8 members) being 1.42 times more likely to be addicted than those from smaller families (≤4 members), suggesting that family dynamics influence addiction risk [23]. This finding is in contrast to our study, where excessive smartphone addiction was more prevalent among students from nuclear families (41.7%) compared to those from non-nuclear families (31.6%). One possible explanation is that students living in nuclear families may experience less supervision, fewer shared responsibilities, and reduced social interaction within the family, which could increase reliance on smartphones for engagement and support. Conversely, individuals from larger or extended families may have more structured routines and greater interpersonal interactions, which might serve as protective factors against excessive smartphone use. Similarly, another study from India found a significantly higher prevalence of smartphone addiction in nuclear families (37%) compared to joint families (24.1%). This difference was attributed to reduced parental supervision and greater independence in nuclear families, which may lead to increased smartphone use [24]. A study conducted among Spanish young adults identified insecure attachment styles and dysfunctional family dynamics as significant predictors of problematic smartphone use. The findings suggested that poor family relationships, which contribute to increased anxiety and fear of missing out, serve as a potent risk factor for smartphone addiction [25].

Finally, nighttime exposure to blue light was associated with prolonged screen time and reduced sleepiness, potentially reinforcing addictive behaviors. A randomized, double-blind, crossover trial among healthy adults reported that nighttime exposure to blue light increased alertness and reduced sleepiness, potentially prolonging screen time and reinforcing addictive behaviors [26]. A meta-analysis including more than 36,000 participants confirmed that pre-sleep smartphone use was associated with poor sleep quality and delayed sleep onset, with a 2.28 times higher risk of sleep disturbance [27]. The alertness induced by blue light may contribute to extended smartphone use, increasing the risk of addiction, whereas blue light-filtered devices may help mitigate these effects.

Strengths and limitations

A key strength of this study is the inclusion of a large and diverse sample of 1,067 college students, along with the use of a standardized and validated tool to assess smartphone addiction. A single investigator collected all data for consistency. Despite its strengths, the study has a few limitations. The cross-sectional study design limits the ability to infer causality between smartphone addiction and its associated factors. Reliance on self-reported data may introduce biases such as social desirability and recall bias. Excluding individuals with pre-existing psychiatric conditions limits the understanding of how addiction interacts with mental health vulnerabilities. The study did not explore psychological outcomes, which could help better understand how smartphone addiction affects mental well-being. The absence of objective usage metrics, such as screen time data, may affect the accuracy of reported smartphone use.

## Conclusions

The present study demonstrates a high prevalence of smartphone addiction (39.9%) among college students aged 18-23 years in Kerala, India, with significant associations with socio-demographic factors and smartphone usage patterns. Addiction was more common among Engineering students, hostel residents, those from nuclear families, and students engaging in late-night smartphone use, particularly after midnight and without the use of blue light filters. These findings highlight the urgent need for public health initiatives promoting digital literacy, healthy smartphone habits, and sleep hygiene among young adults. Targeted interventions, including awareness programs, counseling, and the promotion of sleep-friendly smartphone practices, could help mitigate addiction.

Recommendations include implementing digital hygiene education programs in colleges to raise awareness about the negative effects of excessive smartphone use. Campaigns should encourage reduced screen time before bedtime, ideally avoiding smartphone use at least 30 minutes before sleep, and promote the use of blue light filters at night. Collaborations with app developers to create tools that monitor and limit nighttime smartphone use may also be effective. Future longitudinal studies are recommended to explore causal relationships and evaluate the effectiveness of such interventions. Ongoing research and periodic evaluation of digital behavior policies are essential to adapt strategies and ensure sustainable student well-being.

## Appendices

**Table 5:** Section 1: Socio-demographic variables and smartphone usage pattern (1-18)

Sl. no.	Question	Options
1	Roll number	________
2	Course and branch	________
3	Age in completed years	________
4	Gender	☐ Male
		☐ Female
		☐ Others
5	Religion	☐ Hindu
		☐ Muslim
		☐ Christian
6	Marital status	☐ Single
		☐ Married
		☐ Separated
7	Way of living	☐ Hosteler
		☐ With family
8	Socioeconomic status	☐ Above poverty line (APL)
		☐ Below poverty line (BPL)
9	Type of family	☐ Nuclear family
		☐ Joint family
		☐ Three-generational family
10	Do you own a smartphone?	☐ Yes
		☐ No
11	Age of onset of smartphone use (in years)	____________
12	On average, how many hours do you spend using your smartphone each day?	☐ Less than 1 hour
		☐ 2 hours
		☐ 3 hours
		☐ 5-6 hours
		☐ 7-8 hours
		☐ More than 8 hours
13	Do you think you have a smartphone addiction?	☐ Yes
		☐ No
14	What do you use your smartphone the most for? (multiple options)	☐ Reading
		☐ Songs
		☐ Messaging
		☐ Internet
		☐ Browsing
		☐ Phone calls
		☐ Social media
		☐ Watching videos
15	Do you use your smartphone right before sleeping?	☐ Yes
		☐ No
16	What is the latest time you use your smartphone before going to sleep?	☐ Before 11:00 pm
		☐ 11:00 pm
		☐ 11:30 pm
		☐ 12:00 am
		☐ 12:30 am
		☐ 1:00 am or later
17	How long before going to sleep do you stop using your smartphone?	☐ ˂30 minutes
		☐ 30 minutes to 1 hour
		☐ ˃1 hour
18	Do you use your mobile phone for at least 30 minutes after turning off the lights without using a blue light filter?	☐Yes
		☐ No

**Table 6:** Section 2: MUST questionnaire (19-36) Source: Sharma et al. [15] (permission to use the MUST questionnaire for this study was obtained from Dr. Manoj Kumar Sharma, the corresponding author who authorized the use of the licensed tool for this research purpose) MUST: Mobile Phone Use Screening Test

Sl. no.	Sample item	Never	Rarely	Occasionally	Frequently	Always
19	I start my day with the use of a smartphone	☐	☐	☐	☐	☐
20	I wish I had my smartphone in my hand round the clock	☐	☐	☐	☐	☐
21	I am uncomfortable without my smartphone	☐	☐	☐	☐	☐
22	I can manage the day without my smartphone	☐	☐	☐	☐	☐
23	I experience problems in school, college, or work due to excessive use of smartphones	☐	☐	☐	☐	☐
24	I experience problems in college/workplace due to the usage of smartphone	☐	☐	☐	☐	☐
25	I experience problems in relationships due to my preoccupation with smartphone	☐	☐	☐	☐	☐
26	I can reduce the usage of my smartphone	☐	☐	☐	☐	☐
27	I experience sleep disturbance due to late-night usage of smartphone	☐	☐	☐	☐	☐
28	Reduction of battery power on my smartphone disturbs me	☐	☐	☐	☐	☐
29	Whenever I have a smartphone with me, it is difficult to stop using it	☐	☐	☐	☐	☐
30	I indulge in smartphone usage for longer than earlier	☐	☐	☐	☐	☐
31	I use a smartphone to feel good	☐	☐	☐	☐	☐
32	Smartphone use helps me to overcome my stress	☐	☐	☐	☐	☐
33	I feel more confident while using my smartphone	☐	☐	☐	☐	☐
34	I use my smartphone as the only enjoyable activity of the day	☐	☐	☐	☐	☐
35	When I can't use my smartphone, I feel like I have lost my connection with others	☐	☐	☐	☐	☐
36	I use smartphones to escape or avoid day-to-day problems	☐	☐	☐	☐	☐
